# Neurofibromatosis

**DOI:** 10.3390/cancers12102851

**Published:** 2020-10-02

**Authors:** Koos E. Hovinga, Yasin Temel

**Affiliations:** Department of Neurosurgery, Maastricht University Medical Center, 6202 AZ Maastricht, The Netherlands; y.temel@mumc.nl

In this Special Issue of *Cancer*, a series of 10 papers (seven papers, three reviews) on Neurofibromatosis is presented by international leaders in this field of research.

There are two types of neurofibromatosis. Neurofibromatosis type I (NF-1) is one of the most frequently inherited genetic conditions and is characterized by a monogeneic tumor-predisposition syndrome creating a wide variety of cognitive and behavioral abnormalities. NF-1 patients are at risk to form a number of tumors, such as neurofibromas, optic pathway gliomas and other tumors of the central nervous system. Especially the cognitive development in children with this condition is of great concern. In a study by Taddei et al., the effect of having an additional diagnosis of a brain tumor on the cognitive outcome in children with NF1 was examined. They found that, when corrected for the effect of surgery and chemo-radiation, the co-diagnosis of a brain tumor in children with NF1 could worsen the cognitive and emotional outcome. This is clinically relevant information in the treatment of these patients and confirms the need to systematically evaluate these children for cognitive and emotional problems [1]. When focusing on the risk of developing optic pathway gliomas (OPG), Melloni et al. investigated the genotype of children with NF1 looking at specific sites/types of the NF1 mutations. In their large cohort of 309 NF1 patients, of which 133 patients had OPG, they could not find a significant correlation between the site/type of NF1 mutation and the risk of OPG. They suggest that other modifying influences, such as modifier genes and epigenetic and environmental factors are likely to be involved in determining the NF1 phenotype [2]. This important information gives direction for further research in this field.

Since it is known that women with NF1 under the age of 50 have a fivefold increase in the risk of developing breast cancer, a research group from Toronto (Canada) shared their experience with breast screening in this often impaired emotional functioning population. Of the 61 patients they screened, the worrisome number of four patients were diagnosed with invasive breast cancer, which supports the integration of formal breast-screening programs in the clinical management of NF1 patients [3]. Concluding the section on NF1, Lobbous et al. gave a comprehensive review in their update on NF1-associated gliomas. Importantly they discuss recent notable advances in the treatment of NF-1 associated gliomas, such as clinical trials and collaborative efforts [4].

Neurofibromatosis type II (NF2) is an autosomal dominant tumor disposition disorder caused by the inactivation of the NF2 tumor suppressor gene and the functional loss of its protein product merlin. The typical hallmarks of the disease are the occurrence of bilateral vestibular schwannomas (VS) and in additions the occurrence of multiple nervous system lesions, including meningioma’s, schwannomas and ependymomas. Hearing preservation is a major goal in the treatment of NF2 associated VS, especially in children and adolescents. One original article looked at their hearing preservation results after treatment (comparing surgery vs. no surgery) in a follow up period of up to 167 months. They found that brain stem auditory evoked potential (BAEP) guided surgery did not cause additional hearing loss in the long term and seemed to slow down hearing deterioration in the more aggressive tumors, suggesting that BAEP guided surgery is a safe procedure and can possibly prevent accelerated hearing loss [5].

Since the efficacy of radiosurgery for NF2 associated vs. remains debatable, Shinya et al. looked at the long-term outcome of radiosurgery for sporadic and VS2 related VSs. Patients had a mean follow up of 121 months and in this period no difference in radiation-induced adverse events between the cohorts was found. Excellent local control was found and the authors conclude that, based on these results, radiosurgery is a favorable therapeutic option for NF2 patients with small to medium VS [6]. Despite the efficacy of radiosurgery for the majority of VSs, there is a subset of NF2 related VSs that are radio resistant and systemic treatment is rarely used in these patients. Gugel et al. investigated molecular alterations after radiation in three NF2-associated and five sporadic recurrent VSs after primary radiation. They found contributions of the mTOR and PTEN pathways to radioresistance in these tumors and suggest that particularly mTOR inhibition might be a promising therapeutic strategy [7].

Since hearing-preserving partial resection of NF2 associated VS is the preferred management strategy in modern day medicine, tumor remnants are a logical consequence. These tumor remnants often do grow at one point and, particularly in children and adolescents, can lead to continued hearing deterioration. Adjuvant bevacizumab (a vascular endothelial growth factor monoclonal antibody) is considered an option to slow down this process. One original article found in 16 postoperative patients heterogeneous effects of this treatment with no clear effect in treatment periods compared with periods off treatment. This led the authors to conclude that tumor residuals respond differently to bevacizumab than non-operated faster growing VS [8]. Further research in therefore clearly needed before recommendations for postoperative bevacizumab can be made.

This Special Issue is completed with two reviews. Our group gives an up to date overview with modern day treatment strategies for sporadic and NF2 related VS. One clear point we tried to make is that the management strategy has shifted towards preserving function as longs as possible in combination with tumor control [9]. The other review by Lee et al. focuses on the loss of Merlin/NF2 in meningioma biology. In this comprehensive article they summarize recent findings in the field and provide future directions toward potential therapeutics for this tumor [10].

One direction for further research in this field should focus on finding relevant preclinical models that will allow us to test our hypotheses in laboratories before testing them on patients. In many tumors, great progress has been made in this area and the organoid model is one promising strategy [11]. Care should be taken to thoroughly test these models for clinical relevance before attributing to much value to the results.

Besides the tumor biological approach, another promising approach is radiomics. Radiomics has proven its value in certain areas of cancer while in VS very little is known about is value. One reason is the relatively low number of VSs when compared to glioblastoma, for instance. Multicenter cohorts are required to obtain data from high number of VS patients for radiomics. This line of research will help us eventually to advise our patients better. Being able to predict the growth rate of VS for individual patients based on the quantitative MR parameters is the way to go for better management of the patients.

Let us conclude with a quote from Paul Anderson: “I have yet to see any problem, however complicated, which, when you looked at it the right way, did not become still more complicated”. I think this particularly applies to Neurofibromatosis.
